# Parental psychological control and learning engagement of art college students: exploring the chain-mediated effects of frustration and coping efficacy

**DOI:** 10.3389/fpsyg.2025.1669771

**Published:** 2025-11-21

**Authors:** Jie Shen

**Affiliations:** Anhui Technical College of Mechanical and Electrical Engineering, Wuhu, China

**Keywords:** parental psychological control (PPC), learning engagement, frustration, coping efficacy, art major college students

## Abstract

**Objective:**

This study aims to explore the association between parental psychological control (PPC) and learning engagement in art college students, and examine the chain mediation function of frustration as well as coping efficacy.

**Methods:**

The research involved 612 Chinese art college students, who filled out surveys concerning parental psychological control (PPC), learning engagement, frustration, and coping efficacy.

**Results:**

Path analysis revealed that parental psychological control (PPC) was inversely related to learning engagement among art college students, with frustration and coping efficacy playing a chain-mediated role in this relationship.

**Conclusion:**

This research uncovers the underlying mechanism linking parental psychological control (PPC) to learning engagement among art college students. This offers a reference for educational counseling in colleges and universities as well as families to enhance college students’ learning engagement.

## Introduction

Learning engagement specifically refers to a sustained state characterized by positive emotions that learners display throughout the learning process (Pike et al., 2011). This state is reflected not only in external behaviors—such as classroom participation and task completion—but also in deep psychological engagement, including intrinsic enthusiasm for knowledge exploration and persistence when facing challenges. Its level is directly associated with the quality of learning outcomes and the long-term potential of an individual’s academic development (Kuh, 2001). For this reason, learning engagement has long been a core research topic in education. It serves as both a key indicator of learning quality and a critical variable for predicting academic achievement.

Additionally, numerous studies have demonstrated that the environmental support an individual receives significantly influences the emergence and development of students’ learning engagement (Ryan and Deci, 2000). Among various environmental factors, parental psychological control (PPC)—a crucial dimension of family parenting styles—has garnered considerable attention due to its potential impacts on children’s psychology and behavior (Barber, 1996). Existing research confirms that PPC inhibits children’s autonomous development, which may reduce their learning motivation and self-regulatory abilities. In turn, this exerts a negative influence on both academic performance and learning engagement (Pinquart, 2016).

However, most existing studies have focused on ordinary student groups, with relatively insufficient attention paid to the special group of art college students. Art college students differ from ordinary students in terms of learning content, training models, and psychological traits (Cowdroy and Williams, 2007). Their learning process emphasizes creative thinking and emotional expression more, and they have higher needs for autonomy. PPC may affect their learning engagement through more complex psychological paths (such as frustration and coping efficacy). At the same time, although previous studies have revealed the correlation between PPC and learning engagement (Ng et al., 2014), the internal mechanism between them has not been deeply explored, and there is a lack of systematic analysis of the mediating paths.

In the process of exploring the mechanism between the two, frustration and coping efficacy are two important individual internal psychological factors worthy of attention. Psychological studies have shown that PPC is likely to cause frustration in children (Shek, 2005), and persistent frustration will weaken individuals’ confidence in their own abilities, thereby reducing coping efficacy (Barański and Poprawa, 2023). As a subjective evaluation of one’s ability to handle problems, coping efficacy directly affects the enthusiasm and initiative of learning engagement (Schunk and Zimmerman, 2012). Therefore, there is a close association between frustration and coping efficacy (Chitrakar and Nisanth, 2023). High levels of frustration are often accompanied by low coping efficacy, and the two may form a chain-related relationship between PPC and learning engagement. Nevertheless, there has been a scarcity of research that integrates frustration and coping efficacy into a unified framework to examine their sequential mediating function in the association between PPC and learning engagement.

Building on this, the present study focuses on students from art colleges as its research participants, aiming to systematically examine the serial mediating role of frustration and coping efficacy in the relationship between PPC and learning engagement.

## Background

### PPC and learning engagement

PPC refers to a parenting style in which parents intervene in their children’s psychological activities and emotional development through intrusive means, specifically manifested as excessive control over children’s internal psychological domains such as attachment formation, emotional regulation, and thinking processes (Sabatelli and Mazor, 1985). The core feature of this parenting pattern is the restriction of children’s psychological autonomy, which is fundamentally different from the psychological autonomy-supportive parenting style that focuses on support and understanding and encourages children’s independent exploration (Schaefer, 1965). Barber (1996) pointed out that the implementation paths of PPC mainly include inducing guilt, withdrawing emotional support, expressing disappointment, shaming and punishing, or deliberate neglect, which is essentially a form of “psychological intrusion.” Its purpose is to force children’s cognitive, emotional, and behavioral patterns to conform to parents’ preset standards, which will directly compress children’s self-cognitive space and hinder the formation of their autonomous personality (Barber and Harmon, 2002). For college students in the critical stage of transition from adolescence to adulthood, their construction of self-identity and autonomous needs are at a peak (Zhang et al., 2024). Relevant follow-up studies have confirmed that parents’ explicit behavioral control over their children will gradually weaken during this period, but implicit psychological manipulation will show an upward trend (Rogers et al., 2020). The contradiction between this change in parenting style and college students’ growing autonomous needs is likely to form a sharp conflict, which in turn will have a profound impact on their psychological state and behavioral performance.

Learning engagement, as a core indicator to measure learning quality, is defined as a continuous, positive, and energetic cognitive-emotional integration state that individuals exhibit in the learning process, specifically including three core dimensions: vigor (a sense of energy when facing learning challenges), dedication (a sense of meaning recognition and active investment in learning tasks), and absorption [a state of being immersed in learning and difficult to disengage from Schaufeli et al. (2002)]. This concept is opposite to learning burnout and is a key antecedent variable predicting individual academic achievement, learning persistence, and psychological adaptation (Martin and Borup, 2022).

From the theoretical basis, Self-Determination Theory (SDT) provides core support for analyzing the relationship between PPC and learning engagement (Deci and Ryan, 2008). The theory points out that the satisfaction of the need for autonomy is a key prerequisite for motivating individuals’ proactive behaviors. PPC, through excessive intervention in children’s psychological decision-making processes, is essentially a systematic deprivation of their need for autonomy—parents with high psychological control tend to impose their own will on their children, ignoring their subjective demands, resulting in children being unable to experience behavioral autonomy and a sense of control in key areas such as learning (Schaefer, 1965). This lack of autonomous needs will directly weaken individuals’ intrinsic learning motivation, thereby showing characteristics of low engagement such as decreased vigor, insufficient dedication, and reduced concentration (Ryan and Deci, 2000; Huang, 2020).

In addition, the Ecological Systems Theory also emphasizes that the family microsystem, as the direct environment for individual growth, its interaction quality will shape individual development outcomes through a “bidirectional influence path” (Bronfenbrenner, 1979). As a negative interaction pattern in the family microsystem, PPC not only directly affects college students’ psychological adaptation but also indirectly affects their learning engagement by changing their cognition and coping styles toward the learning environment. For example, individuals who have long been in a high psychological control environment tend to form an “external control” attribution style, which will further reduce their willingness to actively engage in learning (Wijsbroek et al., 2011).

Empirical studies have also provided abundant evidence for the negative correlation between the two (Zhang and Peng, 2025). McArdle and Duda (2004) found that the level of PPC was significantly negatively correlated with children’s learning engagement. Another study showed that high school students who had long been in a family environment with high psychological control were prone to learning burnout after entering college, and the persistence of their learning engagement was significantly lower than that of their peers (Zhao, 2011). A cross-cultural study further points out that the traditional “authoritarian parenting” model in Chinese culture makes parents more inclined to guide their children’s development through psychological manipulation (Ng et al., 2014); in this cultural context, if high psychological control lacks sufficient autonomy support as a balance, it will exert a more significant inhibitory effect on college students’ learning engagement (Wang et al., 2007).

It is worth noting that the learning process of art college students has uniqueness—their professional learning emphasizes creative thinking, personalized expression, and independent exploration ability, which have higher requirements for individuals’ autonomous needs and intrinsic motivation (Samaniego et al., 2024). The compression of autonomous space by PPC may conflict more strongly with the learning characteristics of art majors, thereby causing more prominent negative impacts on their learning engagement. Based on the above theoretical and empirical studies, this study proposes the following hypothesis:

*Hypothesis* 1: PPC is significantly negatively correlated with the learning engagement of art college students.

### The mediating role of frustration and coping efficacy

Frustration refers to the sense of powerlessness or negative emotional experience caused by setbacks in the process of pursuing goals, and it is a comprehensive feeling that includes disappointment, helplessness, and frustration (Gilbert and Allan, 1998). Recent studies have shown that in educational contexts, high levels of PPC continuously compress adolescents’ autonomous space, causing them to frequently encounter obstacles in the process of setting and achieving learning goals, thereby continuously accumulating frustration (Qiang and Jin, 2025). Coping efficacy refers to an individual’s perception and evaluation of their own coping abilities when in a stressful state (Tong, 2005). It largely determines an individual’s attitudes and behaviors when facing learning pressures and challenges. Individuals with high coping efficacy tend to take more active actions to overcome difficulties, while those with low coping efficacy are prone to retreat in the face of predicaments (Zimmerman, 2000).

### The mediating role of frustration

Self-Determination Theory (SDT), proposed by Ryan and Deci (2000), emphasizes that humans possess three fundamental psychological needs: autonomy, competence, and relatedness. When these needs are satisfied, individuals can maintain a positive psychological state and favorable behavioral performance; conversely, hindrance to these needs triggers a series of negative psychological and behavioral outcomes. Excessive PPC impairs individuals’ intrinsic motivation and self-regulatory capacities by restricting their autonomy and disregarding their psychological needs.

Empirical studies have further confirmed this link. For instance, parents’ cold rejection and negative suspicion reduce their children’s sense of accomplishment and provoke doubts about self-worth (Wu and Shen, 2017)—and this sense of powerlessness is a core characteristic of frustration. Shek (2005) longitudinal study on Hong Kong adolescents found that parents’ frequent use of controlling tactics (e.g., emotional withdrawal, conditional love withdrawal) significantly increased their children’s frustrating experiences in academic and daily life. A subsequent cross-cultural study (Shek, 2006) noted that PPC in East Asian cultures is strongly positively correlated with children’s academic frustration, likely due to the erosion of children’s autonomy by high-pressure parenting patterns.

Prior research has also established a close connection between individuals’ experienced frustration and their learning behaviors. When college students encounter obstacles or failures in pursuing academic goals, they may experience frustration and a sense of marginalization, which elicit negative emotions and prevent them from fully engaging in learning (Loder and de Oliveira Wood, 2025). Pekrun et al. (2002) pointed out that academic frustration triggers intense negative emotional experiences in individuals, disrupting their focus and information processing—ultimately lowering their level of learning engagement. A recent study on college students (Loder and de Oliveira Wood, 2025) further verified this: students with high levels of frustration exhibited significantly lower performance in classroom participation, homework completion quality, and exam scores compared to their peers.

Based on the above theoretical and empirical evidence, the following hypothesis is proposed as Hypothesis 2: Frustration plays a mediating role in the relationship between PPC and the learning engagement of art college students.

*Hypothesis* 2: Frustration plays a mediating role between PPC and the learning engagement of art college students.

### The mediating role of coping efficacy

Earlier research has demonstrated a strong link between parental rearing patterns and a person’s coping efficacy. Positive parenting support can significantly enhance children’s coping efficacy, while controlling parenting may have the opposite effect. Social cognitive theory (Bandura, 1986) holds that how a person’s coping efficacy develops is shaped by environmental factors. PPC, by depriving children of opportunities for independent decision-making and trial-and-error, hinders the improvement of their coping confidence and problem-handling capabilities. Parents’ excessive control over their children’s thoughts and emotions is not conducive to shaping their expectations of their ability to cope with unknown events independently (Liang et al., 2019), and this ability expectation is the main embodiment of coping efficacy. Moreover, research has indicated that youngsters from families exerting high psychological control tend to develop “learned helplessness” when confronting challenges (Miller and Seligman, 1975) and lack confidence in dealing with their own learning issues.

At the same time, coping efficacy is one of the important factors affecting individual learning engagement (Deng et al., 2022; Liu et al., 2023; Heo et al., 2021). According to self-efficacy theory, students with high coping efficacy tend to regard learning tasks as conquerable challenges rather than threats, thus taking the initiative to invest more time and energy (Schunk and Zimmerman, 2012). The study by Zhang et al. (2009) confirmed that students’ coping efficacy has a marked positive association with learning engagement, and the two factors of competence and confidence in coping efficacy can significantly predict learning engagement. On this basis, the hypothesis below is put forward:

*Hypothesis* 3: Coping efficacy serves as a mediating factor in the link between PPC and art college students' learning engagement.

### The chain mediating role of frustration and coping efficacy

According to the self-concept theory, an individual’s perception of their own abilities, values, and efficacy significantly influences their emotional experiences and behavioral performance. When an individual’s self-concept is negatively impacted, such as by frequent experiences of setbacks or denial, it is likely to trigger intense feelings of frustration, and this emotion will further weaken their confidence in their own coping abilities (Baumeister, 1999). An individual’s perception of their own failures may lead to a sense of incompetence when dealing with stressful events, resulting in reduced coping efficacy (Bandura and Wessels, 1997). When individuals frequently experience frustration, this negative emotion will continuously strengthen their negative cognition of their own abilities, making them more likely to fall into self-doubt when facing challenges such as learning tasks and artistic creation. They may think that they lack the ability and resources to effectively solve problems, thereby reducing active attempts and efforts, forming a vicious circle of “frustration—negative cognition—low coping efficacy.”

Artistic creation emphasizes the expression of personality, with subjective and diverse evaluation criteria. The uncertainty of whether works can be recognized by different groups makes art major college students more prone to frustration. PPC, such as excessive interference in learning direction and negation of creative achievements, will further aggravate their experience of frustration. When feelings of frustration intensify, college students’ self-assurance and capacity to handle academic challenges tend to diminish (Yang et al., 2024; Zhao et al., 2021). This makes it harder for them to sustain a proactive approach to learning and consistent investment of effort, ultimately resulting in reduced learning engagement (Liu et al., 2024). Thus, the following hypothesis is proposed:

*Hypothesis* 4: Frustration and coping efficacy function as sequential mediators in the connection between PPC and art college students' learning engagement.

### The present study

Existing research has consistently indicated a notable negative correlation between PPC and students’ learning engagement (McArdle and Duda, 2004). This controlling parenting style may inhibit learning motivation by triggering negative psychological experiences. Mechanistically, frustration has been confirmed to act as a mediator in how parenting styles relate to adolescent development—Shek (2005) found that frustration significantly mediates the link between parental parenting behaviors and adolescents’ emotional health. As an important manifestation of academic emotions, learning engagement may also be affected by this path. Meanwhile, as a core psychological resource for individuals to cope with stress, the key role of coping efficacy in alleviating learning pressure and enhancing intrinsic motivation has been confirmed by the research of Bandura and Wessels (1997). Therefore, exploring the mediating role of these two factors will help to more effectively grasp the link between PPC and learning engagement.

**Figure 1:**
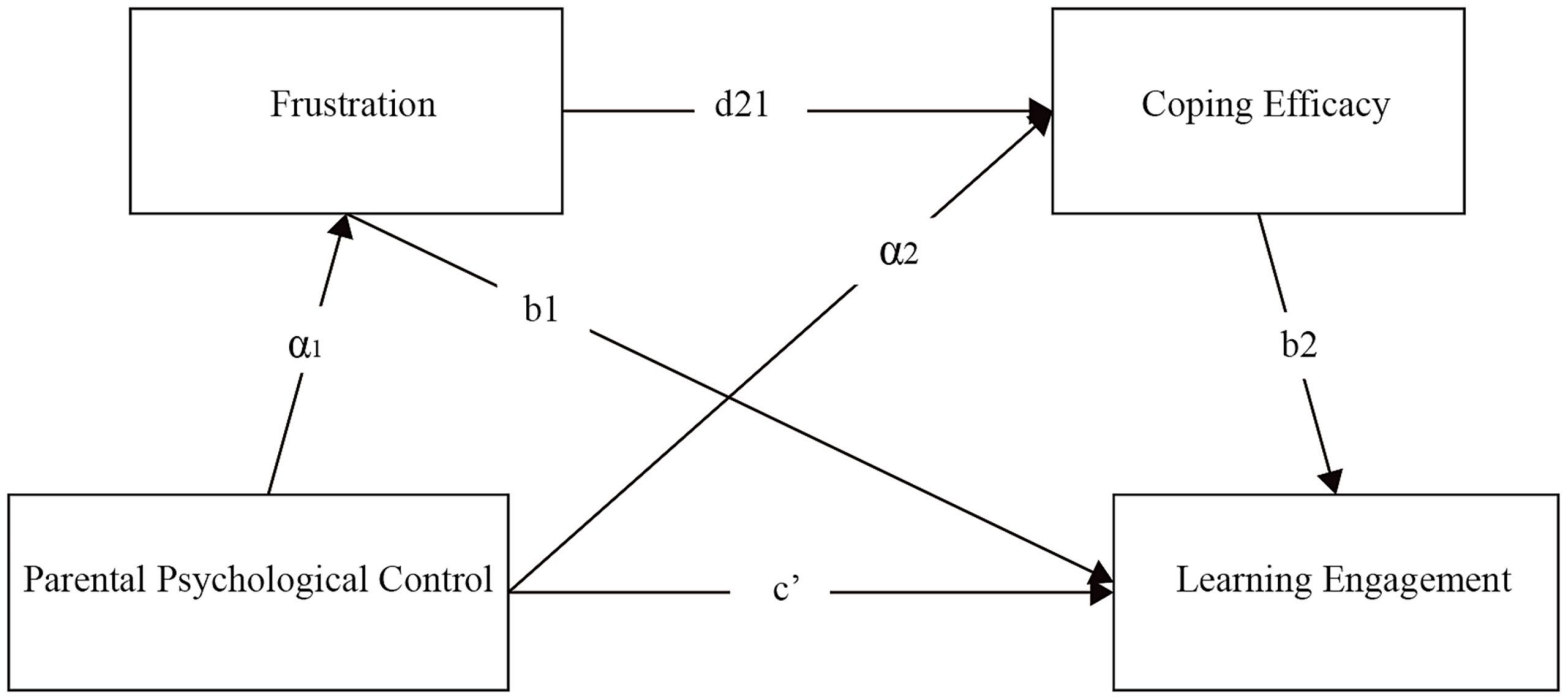
A hypothetical multiple sequential mediation model.

However, prior research has predominantly centered on either the direct association between PPC and learning engagement or the mediating function of an individual variable. While some research has examined the role of frustration in academic challenges (Pekrun et al., 2002) and the positive impact of coping efficacy on academic performance (Schunk and Zimmerman, 2012), no studies have integrated frustration and coping efficacy into a unified framework to investigate their sequential mediating function in the relationship between PPC and art students’ learning engagement. Thus, the current study aims to explore the relationship between PPC and art students’ learning engagement, and to confirm whether frustration and coping efficacy act as sequential mediators in this association. It is anticipated that the results of this research will expand existing understanding of the mechanism underlying the connection between PPC and learning engagement (see Figure 1), and provide new theoretical support for studies in related fields.

## Materials and methods

### Participants and procedure

According to the sampling criteria proposed by Roscoe et al. (2018), for questionnaire surveys focusing on mediating effect models, a sample size of 30–500 is generally sufficient to meet the requirements of statistical power. This study targeted art college students and adopted a multi-stage stratified sampling method to recruit a total of 612 college students from the art colleges of 5 comprehensive universities in China (Anhui Technical College Of Mechanical and Electrical Engineering, School of Art of Anhui Polytechnic University, School of Art of Anhui Normal University, Wuhu Vocational and Technical University, and Hefei University).

The sample composition is as follows: the age range is 18–24 years (M = 19.89, SD = 1.51), among which freshmen account for 22.7% (*n* = 139), sophomores account for 25.4% (*n* = 155), juniors account for 28.9% (*n* = 177), and seniors account for 23.0% (*n* = 141). The majors cover fields such as fine arts (26.2%), music performance (21.5%), dance (18.4%), drama and film (17.8%), and design (16.1%). Female participants account for 64.1% (*n* = 392).

This study adopted a cross-sectional design, and the survey was conducted from March to June 2025. Psychological teachers and postgraduate students in psychology who had received standardized training served as test administrators. They used unified instructions to clarify the research purpose and emphasize principles such as informed consent, confidentiality, and anonymity. Then, group testing was conducted on-site in the study rooms or rehearsal venues of the art colleges, with classes as the unit. The total duration of the testing was 18 min. After the participants completed the questionnaires, the questionnaires were collected on the spot. All questionnaires were presented in Chinese, and the first page indicated the research purpose, anonymity principle, and privacy protection measures. To ensure data quality, all questionnaires were checked for completeness, and invalid responses (such as excessively short response time and regular response patterns) were excluded. Finally, the effective sample size was 612 (with an effective recovery rate of 93.3%, and a total of approximately 656 samples were collected).

The research procedures strictly followed academic ethical norms and have been approved by the Ethics Review Committee of Anhui Technical College Of Mechanical and Electrical Engineering. Participants received cultural and creative products worth 3 yuan (approximately 0.5 US dollars) as a reward after completing the questionnaires.

### Measures

#### Parental psychological control scale

The degree of PPC as perceived by art college students was evaluated using the psychological control subscale from the Chinese adapted version of the Psychological Control Scale (Wang et al., 2007), which was initially developed by Barber (1996). This scale ranks among the most commonly utilized instruments in the domain of family parenting both in China and abroad, and is especially appropriate for gaging adolescents’ perceptions of psychological control. Comprising 18 items, it employs a 5-point Likert scoring system (1 = entirely untrue to 5 = entirely true) and encompasses three dimensions: guilt induction, love withdrawal, and arbitrary authority. Higher scores reflect more intense PPC over their offspring. The scale demonstrates favorable dependability and validity in samples of Chinese university students, with the Cronbach’s *α* coefficient reaching 0.97 in the present study.

#### Frustration scale

The degree of frustration experienced by art college students was assessed using the Frustration Scale, which was initially created by Gilbert and Allan (1998) and subsequently adapted by Tang et al. (2019). This scale has been extensively applied in research across diverse populations, boasting good reliability and validity, and is capable of effectively measuring the frustration individuals feel when confronted with stress and challenges. Comprising 16 items, it includes two dimensions: low sense of achievement and sense of decadence. Employing a 5-level Likert scale (ranging from “1 - totally inconsistent” to “5 - totally consistent”), higher scores indicate a stronger feeling of frustration. In the present research, the Cronbach’s *α* coefficient of this measure was 0.98.

#### Coping efficacy scale

This research employed the Chinese version of the Coping Efficacy Questionnaire, developed by Tong (2005) through adaptations from Bandura (1982). This scale is extensively employed in domestic psychological research within China, demonstrating sound reliability and validity, and can precisely assess individuals’ confidence in and evaluations of their own coping capabilities when encountering stressful circumstances. It comprises three dimensions—cognitive level, confidence level, and perceived competence—including 17 items in total. A 4-level Likert scale is used, with ratings spanning from 1 (completely inconsistent) to 4 (completely consistent); higher scores signify greater individual coping efficacy. Having exhibited favorable reliability and validity across studies with various samples, this measure produced a Cronbach’s *α* of 0.97 in the current research.

#### Learning engagement scale

This research adopted the Academic Involvement Scale adapted by Fang et al. (2008), which originated from the work of Schaufeli et al. (2002). This scale is extensively utilized in educational psychology research, featuring sound reliability and validity, and can comprehensively and precisely gauge students’ degree of involvement in the learning process. It comprises 17 items across three facets: vitality, dedication, and absorption. A 7-level Likert scale is employed, with ratings spanning from “1 - totally disagree” to “7 - totally agree,” and all items are positively scored. Higher scores indicate a stronger degree of learning engagement. In the current study, the Cronbach’s α of this measure yielded a coefficient of 0.98.

## Results

### Preliminary analysis

For the present research, IBM SPSS 26.0 and AMOS 24.0 were employed to perform descriptive statistics, regression analyses, and assessments of sequential multiple mediating effects. To assess the influence of common method bias on the results, Harman’s single-factor test was employed. It was found that four factors with eigenvalues exceeding 1 were extracted in total, and the first factor accounted for 19.82% of the variance—below the 40% cutoff (Podsakoff et al., 2003)—suggesting that no significant common method bias existed in this research.

Table 1 displays the means, standard deviations, and Pearson correlation coefficients of each variable. The mean score of PPC was 2.537 (SD = 1.156), frustration had an average of 2.401 (SD = 1.269), coping efficacy was 1.795 (SD = 0.822), and learning engagement averaged 3.070 (SD = 1.902).

**Table 1:** Pearson correlation coefficients and MSD of each factor (*N* = 612).

Variable	Range	Mean	Standard deviation	1	2	3	4	5	6
1. PPC	1–5	2.537	1.156	1					
2. Frustration	1–5	2.401	1.269	0.482**	1				
3. Coping efficacy	1–4	1.795	0.822	−0.337**	−0.249**	1			
4. Learning engagement	1–7	3.070	1.902	−0.530**	−0.352**	0.571**	1		
5. Age		19.873	1.506	−0.052	0.018	0.075	0.028	1	
6. Gender		0.641	0.480	0.017	0.039	−0.112**	−0.093*	0.025	1

Gender: 0 = Male; 1 = Female.*p < 0.05, **p < 0.01.

Correlation analyses indicated that PPC was significantly and positively linked to frustration (*r* = 0.482, *p* < 0.01), while showing significant negative associations with both coping efficacy (*r* = −0.337, *p* < 0.01) and learning engagement (*r* = −0.530, *p* < 0.01). Additionally, frustration was significantly negatively correlated with coping efficacy (*r* = −0.249, *p* < 0.01) and learning engagement (*r* = −0.352, *p* < 0.01), whereas coping efficacy and learning engagement exhibited a significant positive correlation (*r* = 0.571, *p* < 0.01). These results offer initial support for the directional relationships between variables as proposed in the research hypotheses.

### Hypothesis testing

This study employed multiple regression analysis, with PPC, frustration, coping efficacy, gender (0 = male, 1 = female), and age as predictor variables, to conduct a predictive analysis on the learning engagement of art college students (Table 2).

**Table 2:** Results of multiple regression analysis.

Variables	Model1			Model2			Model3		
	Frustration			Coping efficacy			Learning engagement		
	*β*	SE	*t*	*β*	SE	*t*	*β*	SE	*t*
Constant	0.287	0.611	0.470	1.878	0.421	4.464	3.605	0.783	4.605
Gender	0.078	0.094	0.834	−0.179	0.065	−2.777	−0.136	0.119	−1.143
Age	0.036	0.030	1.205	0.036	0.021	1.724	−0.026	0.038	0.698
PPC	0.531	0.039	13.627	−0.197	0.031	−6.423	−0.574	0.058	−9.901
Frustration				−0.073	0.028	−2.620	−0.110	0.051	−2.143
Coping efficacy							1.002	0.074	13.491
Learning engagement									
R^2^	0.235			0.138			0.461		
F	62.386			24.305			103.712		

*N* = 612; *p < 0.05; **p < 0.01.

First, the direct relationships between PPC and frustration, and between PPC and coping efficacy were tested in Model 1 and Model 2, respectively. The regression outcomes with frustration as the criterion variable indicated that PPC notably and positively forecasted frustration (*β* = 0.531, *p* < 0.001);the regression results with coping efficacy as the dependent variable (Model 2) indicated that PPC notably and negatively forecasted coping efficacy (*β* = −0.197, *p* < 0.001). In Model 3, which included all predictor variables, the combined explanatory power of PPC, frustration, coping efficacy, gender, and age for learning engagement was 46.1% [R^2^ = 0.461, *F* (5, 606) = 103.712, *p* < 0.001]. Among these, PPC, frustration, and coping efficacy were significant predictors, while gender and age did not reach a significant level.

Hayes (2013) PROCESS Model 4 was employed to examine the mediating effects of frustration and coping efficacy in the relationship between PPC and learning engagement. This involved constructing a 95% bias-corrected confidence interval (CI) using 5,000 Bootstrap samples. Where the CI excludes 0, the mediating effect is deemed significant, with findings presented in Table 3.

**Table 3:** Summary of the effect analysis process.

Effect	Effect	SE	*t*	*p*	LLCI	ULCI
Direct effect	−0.570	0.058	−9.849	0.000	−0.684	−0.457
Total effect	−0.872	0.056	−15.435	0.000	−0.983	−0.761
Indirect effect 1	−0.060	0.017	−3.622	0.000	−0.068	−0.003
Indirect effect 2	−0.202	0.025	−8.018	0.000	−0.172	−0.074
Indirect effect 3	−0.039	0.010	−3.955	0.000	−0.044	−0.004
Total indirect effect	−0.302	0.026	−11.441	0.000	−0.234	−0.131

LLCI denotes the lower limit of the 95% confidence interval for the estimated value, while ULCI stands for the upper limit of this interval. path1: PPC → Frustration → Learning engagement; path 2: PPC → Coping efficacy → Learning engagement; path 3: PPC → Frustration → Coping efficacy → Learning engagement.

**Figure 2:**
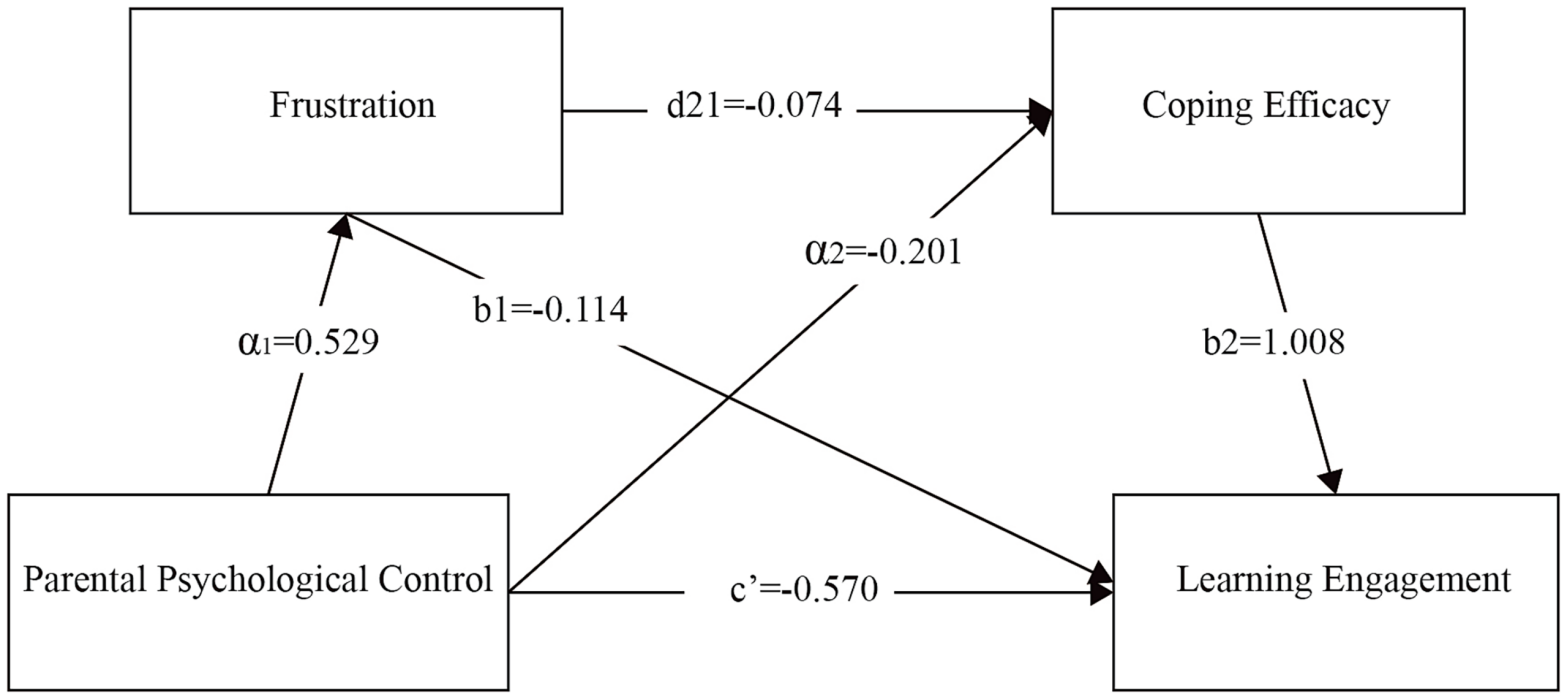
Path analysis model of PPC, frustration, coping efficacy, and learning engagement.

The structural equation model in Figure 2 illustrates four paths between PPC and learning engagement: (1) the direct effect path c’; (2) PPC → frustration → learning engagement (a1b1); (3) PPC → coping efficacy → learning engagement (a2b2); (4) PPC → frustration → coping efficacy → learning engagement (a1d21b2). The lines in the diagram denote pathways, with a1, a2, b1, b2, d21, and c’ standing for path coefficients.

As shown in Table 3, PPC is significantly related to learning engagement (c’ = −0.570, *p* < 0.001). PPC exerts an indirect impact on learning engagement via frustration, with the effect value being −0.060 (95% CI = [−0.068, −0.003]). Its indirect effect through coping efficacy is −0.202 (95% CI = [−0.172, −0.074]), and the sequential mediating effect through frustration and coping efficacy in turn is −0.039 (95% CI = [−0.044, −0.004]). Since none of the confidence intervals for these three mediating pathways include 0, it suggests that both the individual mediating effects and the chain mediating effect proposed in the hypotheses are statistically significant. Thus, Hypotheses 1, 2, 3, and 4 are all validated.

Further analysis of the magnitude of mediating effects revealed that the single mediating effect of coping efficacy (−0.202) was significantly greater than both the single mediating effect of frustration (−0.060) and the chain mediating effect (−0.039; Δβ = 0.142, *p* < 0.01). This indicates that among the influencing paths of PPC on learning engagement, the mediating role played by coping efficacy is the most prominent.

## Discussion

Guided by Self-Determination Theory, Social Cognitive Theory, Self-Efficacy Theory, and Self-Concept Theory, this study explores the relationship between PPC and learning engagement among college students in art schools, and analyzes the chain mediating role of frustration and coping efficacy. The results show that: PPC is significantly negatively correlated with learning engagement of art school students; frustration and coping efficacy each play a significant mediating role between them; and the chain mediating effect of “frustration → coping efficacy” is also statistically significant. The above results verify all hypotheses of this study, and the specific discussion of each hypothesis is as follows:

Hypothesis 1 proposes that “PPC is significantly negatively correlated with learning engagement of college students in art schools.” This hypothesis is fully supported and consistent with the conclusions of McArdle and Duda (2004) and Pinquart (2016), further confirming the cross-group stability of the negative impact of PPC on learning engagement. A study by Zhang and Peng (2025) on 1,725 Chinese children (Mage = 10.77 years old) also points out that PPC significantly reduces children’s learning engagement, forming a “cross-age stage” verification with this study. It suggests that this negative impact may have preliminary developmental continuity from childhood to early adulthood (such as the group of college students in art schools in this study). Notably, Zhang and Peng (2025) also found that PPC simultaneously reduces life satisfaction. In this study, the self-doubt of art school students caused by the deprivation of creative autonomy (Cowdroy and Williams, 2007) essentially reflects the implicit damage to life satisfaction. Together, they reveal the negative impact of PPC on individuals in the dual domains of “academic - life.”

From the perspective of Self-Determination Theory (Ryan and Deci, 2000; Deci and Ryan, 2008), college students are in a critical stage of transition from adolescence to adulthood, and their need for autonomy is significantly enhanced. Moreover, the learning of college students in art schools emphasizes independent thinking and creative expression more, and their needs for creative thinking and emotional expression are higher than those of ordinary college students. However, PPC (such as guilt induction, love withdrawal, and arbitrary authority; Barber, 1996; Barber and Harmon, 2002) will directly invade their psychological space, deprive them of independent decision-making power in learning and creation, and lead to the failure to meet the need for autonomy. This parenting style will not only aggravate parent–child conflicts in the process of “separation - individuation” (Wijsbroek et al., 2011), but also make students pay excessive attention to parental approval rather than the internal pleasure of art learning. When their creative works or learning choices are denied by their parents, students are prone to inner pain and self-doubt, which in turn leads to a significant decline in the core dimensions of learning engagement (vitality, dedication, and concentration; Schaufeli et al., 2002). This direct effect indicates that PPC has a “source nature” on the impact of learning engagement. Relevant interventions need to start from the core contradiction of family upbringing (deprivation of autonomy) to fundamentally reduce its negative effect and lay a foundation for subsequent intervention in mediating paths.

Hypothesis 2 proposes that “frustration plays a mediating role between PPC and learning engagement of college students in art schools.” The data results support this hypothesis and are consistent with the research logic that “parenting style affects individual academic adaptation through negative emotions” (Shek, 2005, 2006). A study by Loder and de Oliveira Wood (2025) confirms that high frustration reduces college students’ academic performance, which directly echoes the conclusion of this study that “frustration inhibits learning engagement.” This study further clarifies the antecedent path of “PPC → frustration” and supplements the “family source” mechanism of frustration. Previously, a study by Heo et al. (2021) on online learning during the epidemic found that difficulties encountered by individuals in technology use and time management indirectly affect learning engagement through negative emotions. Although the scenario focuses on online learning, the logical chain of “external pressure → negative emotions → decreased engagement” is consistent with this study, indicating that the mediating role of frustration has “scenario universality.” This study focuses on the “creative pressure” scenario of college students in art schools, further enriching the research on the group specificity of the mediating mechanism of frustration.

From the perspective of Self-Determination Theory, PPC hinders the satisfaction of the need for autonomy, which will damage students’ sense of self-worth and competence (Wu and Shen, 2017), making them feel powerless to “achieve learning and creative goals,” thereby triggering a strong sense of frustration (Gilbert and Allan, 1998). Academic frustration will trigger strong negative emotional reactions, interfere with cognitive processing ability, reduce learning concentration, and make it difficult to maintain a high level of learning participation (Pekrun et al., 2002). This mediating effect indicates that PPC affects learning engagement through the “emotional path.” It is necessary to target the causal chain of “parental control → frustration” to alleviate students’ frustration experience caused by parental intervention from the source and prevent negative emotions from further transmitting to the cognitive level.

Hypothesis 3 proposes that “coping efficacy plays a mediating role between PPC and learning engagement of college students in art schools.” The research results support this hypothesis and are consistent with the core view of Social Cognitive Theory (Bandura, 1986) that “environmental factors shape self-efficacy,” while verifying the key role of coping efficacy in academic adaptation (Schunk and Zimmerman, 2012). The “coping efficacy” in this study is essentially the specific manifestation of self-efficacy in the field of “problem coping,” which is highly consistent with the relevant conclusions of “self-efficacy mediates academic engagement.” A study by Deng et al. (2022) on 926 high school students found that individuals with high self-efficacy are more likely to convert positive motivation into learning engagement, which is consistent with the logic of “high coping efficacy → high learning engagement” in this study. This study further clarifies the negative path of “PPC → decreased coping efficacy” and supplements the “inhibitory source” of self-efficacy. In addition, a study by Liu et al. (2023) on 466 college students confirms that teacher support indirectly promotes learning engagement by improving self-efficacy. From the perspective of “family negative factors,” this study reveals that PPC inhibits engagement by reducing coping efficacy. Together, they form a “two-way verification” of “supportive factors → self-efficacy → increased engagement” and “controlling factors → coping efficacy → decreased engagement,” improving the mechanism of self-efficacy in academic engagement.

Social Cognitive Theory holds that coping efficacy is mainly formed through the accumulation of life experience and the guidance of the external environment. However, PPC will deprive college students in art schools of the opportunity to face and solve learning and life problems independently (Liang et al., 2019), making them develop “learned helplessness” and leading to a decline in coping efficacy (Miller and Seligman, 1975). According to Self-Efficacy Theory (Bandura, 1982; Schunk and Zimmerman, 2012), individuals with high coping efficacy tend to regard learning and creative challenges as “solvable problems” and take the initiative to invest time and energy to deal with them. Individuals with low coping efficacy are prone to avoid difficulties, leading to a decline in learning engagement. This mediating effect reveals the “cognitive path” through which PPC affects learning engagement, that is, reducing coping efficacy by limiting independent growth opportunities, thereby reducing learning engagement. This suggests that the intervention of this cognitive path needs to focus on “enhancing the perception of coping ability,” and offset the weakening effect of parental control by supplementing independent experience and strengthening ability recognition.

Hypothesis 4 proposes that “frustration and coping efficacy play a chain mediating role between PPC and learning engagement of college students in art schools.” The research results support this hypothesis. This chain path further reveals the multi-level mechanism of “emotion - cognition” linkage between PPC and learning engagement, enriching the in-depth understanding of the relationship between them. Previously, a study by Zhang and Peng (2025) proposed that “perceived social mobility” is a “resilience factor” to alleviate the negative impact of PPC. Although it focuses on “cognitive adjustment variables,” it provides a new perspective for understanding the chain mechanism of this study. In this study, “coping efficacy” is the “cognitive buffer resource” within the individual, and “perceived social mobility” is the “cognitive adjustment resource” for external environment perception. Together, they indicate that individual cognitive variables are the key to blocking the negative transmission of PPC. In addition, Deng et al. (2022) found the chain path of “self-efficacy → positive emotions → learning engagement,” while this study reveals the negative chain path of “frustration → coping efficacy → learning engagement.” Together, they form a complement to the “positive - negative” chain mechanism, improving the research on the linkage of “emotion - cognition - behavior.”

From the perspective of Self-Concept Theory (Baumeister, 1999), individual emotional experience is closely related to self-cognitive evaluation: PPC first induces frustration (Shek, 2005), and persistent negative emotions will damage the self-concept, leading individuals to form negative cognitions about their own abilities (Bandura and Wessels, 1997). Specifically, for college students in art schools, the frequent frustration caused by PPC will make them gradually doubt their ability to cope with learning and creative challenges, thereby reducing coping efficacy. The decline in coping efficacy will further inhibit learning engagement (Liu et al., 2024; Yang et al., 2024). It should be noted that college students in art schools face the unique situation of “high uncertainty of creative results and diverse evaluation criteria” (Cowdroy and Williams, 2007), which makes them more prone to frustration. The chain effect of “frustration → coping efficacy” will make the negative impact of PPC more lasting and far-reaching. This chain mediating path indicates that the impact of PPC on the learning engagement of college students in art schools is a complex process of “emotional trigger - cognitive deterioration - behavioral withdrawal.” It suggests that interventions need to take into account the coordination of “emotional source” and “cognitive transmission,” and form a dual intervention idea of “emotional regulation + cognitive empowerment” to completely break the negative chain reaction.

To clarify the relative importance of the three indirect paths, this study further compared the effect sizes of each mediating effect. The results show that the independent mediating effect of coping efficacy (−0.202) is significantly greater than the independent mediating effect of frustration (−0.060) and the chain mediating effect (−0.039), indicating that among the three indirect paths, the mediating role of coping efficacy is the most prominent. From the perspective of Self-Efficacy Theory (Bandura, 1982; Zimmerman, 2000), as a subjective evaluation of an individual’s own coping ability, coping efficacy directly affects their behavioral choices and investment level when facing challenges. Frustration, as an emotional experience, can only indirectly affect learning engagement by influencing cognition (such as coping efficacy). Therefore, the predictive effect of coping efficacy on learning engagement is more direct and powerful. This finding provides a key basis for setting intervention priorities: compared with the regulation of frustration at the emotional level, improving coping efficacy may be a more core direction to alleviate the negative impact of PPC, which needs to be focused on in subsequent practical interventions.

### Theoretical significance of the study

This study makes the following contributions at the theoretical level. First, it enriches the group-specific application of Self-Determination Theory. Previous studies mostly focused on ordinary students. This study, targeting the “high demand for autonomy” of college students in art schools, confirms that the deprivation of their autonomy by PPC will directly inhibit learning engagement. It provides new evidence for the adaptability of this theory in special education groups and refines the group difference mechanism of “basic psychological needs satisfaction → academic adaptation.” Second, it improves the “emotion - cognition” dual-path integration framework. Through the chain mediating effect of “frustration → coping efficacy,” this study reveals the systematic process of “emotional trigger - cognitive transmission - decreased engagement,” clarifies the core mediating role of coping efficacy, and supplements the multi-dimensional chain of “family negative parenting → academic behavior.” Third, this study confirms that PPC reduces coping efficacy by depriving independent opportunities, clarifies the “inhibitory source” of self-efficacy, and explains the transformation of “frustration → coping efficacy” in combination with Self-Concept Theory. It provides empirical support for the application of this theory in the emotion - cognition connection in the academic field.

### Practical significance of the study

For families, parent classes and online micro-courses can be used to help parents of art students understand the contradiction between “psychological control and artistic autonomy,” clarify that behaviors such as guilt induction and arbitrary evaluation will deprive creative decision-making power and lead to children’s self-doubt. It guides them to adopt supportive parenting, such as respecting artistic preferences, providing emotional support when facing creative bottlenecks, and paying attention to the dual impact of parenting styles on “academic - life.” For art schools, it is necessary to build a two-dimensional academic support system targeting the “emotion - cognition” path. At the emotional regulation level, courses on “creative frustration management” can be offered, mindfulness stress reduction techniques can be taught, and a green channel for psychological counseling can be established. At the cognitive level, “coping efficacy improvement” training (such as creative task decomposition and ability affirmation workshops) can be carried out to supplement independent growth experience.

### Research limitations and future research directions

The present research is subject to several limitations. First, the sample, selected via convenience sampling from eastern Chinese art colleges, may suffer from selection bias, limiting the generalizability of results to the national art student population. Second, the cross-sectional research design and dependence on self-reported measures (such as the PPC Scale and Learning Engagement Scale) impede the establishment of causal links and might give rise to common method bias (e.g., social desirability effects). Third, only the chain mediating role of frustration and coping efficacy was explored, with no consideration of moderators (e.g., art major-specific factors like creative pressure, family artistic support) or distinctions between dimensions of PPC (e.g., love withdrawal, guilt induction) in mediating paths.

To address these limitations, future research could: (1) Use multi-stage random sampling to include art students from diverse regions and majors, balance demographic factors, adopt longitudinal designs (e.g., 6-month interval three-wave measurements) with cross-lagged models, and integrate multi-source data (parental reports, teacher evaluations) to reduce bias. (2) Incorporate variables like “artistic autonomy needs” and “perceived professional pressure” to explore indirect effects via inhibited artistic identity; test moderators such as family artistic support (e.g., high support may weaken frustration’s mediating role) and tutor guidance styles. (3) Conduct cross-group comparisons (art vs. non-art students, different art majors) and Sino-Western cultural comparisons to examine cultural specificity in psychological control’s impact. (4) Revise the PPC Scale with localized items (e.g., “interference in artistic choices”) to better reflect art students’ family interactions.

## Conclusion

This study confirms that PPC affects the learning engagement of art college students through three paths: first, a direct negative impact; second, separate mediation through frustration or coping efficacy; third, chain mediation through “frustration → coping efficacy,” among which the mediating role of coping efficacy is the most prominent. This finding not only verifies the chain mediating model of “PPC → frustration → coping efficacy → learning engagement” but also reveals the internal mechanism of family parenting styles on the academic development of art students. Especially in the context of the unique creative pressure and self-expression needs of art majors, PPC may have a persistent negative impact on their learning engagement by disrupting the balance of students’ psychological resources. This study provides a fresh viewpoint on exploring the link between family factors and art college students’ learning engagement, confirms the mediating significance of frustration and coping efficacy, and offers empirical evidence for optimizing parent–child interaction patterns and improving students’ psychological resilience in educational practice.

## Data Availability

The original contributions presented in the study are included in the article/supplementary material, further inquiries can be directed to the corresponding author.
